# Exercise in claudicants increase or decrease walking ability and the response relates to mitochondrial function

**DOI:** 10.1186/s12967-017-1232-6

**Published:** 2017-06-07

**Authors:** Michel van Schaardenburgh, Martin Wohlwend, Øivind Rognmo, Erney J. R. Mattsson

**Affiliations:** 1 Department of Circulation and Medical Imaging, Norwegian University of Science and Technology, PO box 8905, 7491 Trondheim, Norway; 2 Department of Vascular Surgery, St. Olavs Hospital, Trondheim, Norway

**Keywords:** Intermittent claudication, Exercise, Mitochondria, Preconditioning, Ischemia reperfusion injury

## Abstract

**Background:**

Exercise of patients with intermittent claudication improves walking performance. Exercise does not usually increase blood flow, but seems to increase muscle mitochondrial enzyme activities. Although exercise is beneficial in most patients, it might be harmful in some. The mitochondrial response to exercise might therefore differ between patients. Our hypothesis was that changes in walking performance relate to changes in mitochondrial function after 8 weeks of exercise. At a subgroup level, negative responders decrease and positive responders increase mitochondrial capacity.

**Methods:**

Two types of exercise were studied, calf raising and walking (n = 28). We wanted to see whether there were negative and positive responders, independent of type of exercise. Measurements of walking performance, peripheral hemodynamics, mitochondrial respiration and content (citrate synthase activity) were obtained on each patient before and after the intervention period. Multiple linear regression was used to test whether changes in peak walking time relate to mitochondrial function. Subgroups of negative (n = 8) and positive responders (n = 8) were defined as those that either decreased or increased peak walking time following exercise. Paired *t* test and analysis of covariance was used to test changes within and between subgroups.

**Results:**

Changes in peak walking time were related to changes in mitochondrial respiration supported by electron transferring flavoprotein (ETF + CI)_P_ (p = 0.004), complex I (CI + ETF)_P_ (p = 0.003), complex I + complex II (CI + CII + ETF)_P_ (p = 0.037) and OXPHOS coupling efficiency (p = 0.046) in the whole group. Negative responders had more advanced peripheral arterial disease. Mitochondrial respiration supported by electron transferring flavoprotein (ETF + CI)_P_ (p = 0.0013), complex I (CI + ETF)_P_ (p = 0.0005), complex I + complex II (CI + CII + ETF)_P_ (p = 0.011) and electron transfer system capacity (CI + CII + ETF)_E_ (p = 0.021) and OXPHOS coupling efficiency decreased in negative responders (p = 0.0007) after exercise. Positive responders increased citrate synthase activity (p = 0.010).

**Conclusions:**

Changes in walking performance seem to relate to changes in mitochondrial function after exercise. Negative responders have more advanced peripheral arterial disease and decrease, while positive responders increase mitochondrial capacity.

*Trial registration* ClinicalTrials.gov ID: NCT023110256

## Background

Mitochondria play a central role in the muscular response to exercise and are depended on oxygen for the energy production. Patients with intermittent claudication (IC) have a reduced blood flow to their extremities and thereby reduced oxygen supply to the muscles. Patients with IC have in addition been shown to have mitochondrial dysfunction [1, 2]. This indicates that the oxygen still delivered will not be optimally utilized. Exercise of patients with IC seems to increase walking ability, without changing blood flow [3], but rather increase mitochondrial enzyme activity [4]. Exercise induces ischemia and reperfusion starts at rest. It has been imposed that repetitive cycles of exercise followed by rest may be important for increased walking ability [5–7]. This is equal to induce a state of preconditioning that stimulates the mitochondria [8, 9]. There is simultaneously a possibility that exercise might damage muscle mitochondria [10, 11].

A number of patients with IC do not seem to respond to exercise [12]. It seems as if there are both responders and non-responders or even negative responders among these patients.

The aim of this investigation was to explore whether changes in walking performance could be explained by changes in mitochondrial function. Furthermore, we wanted to identify patients with IC being negative or positive responders after exercise and to clarify potential differences in the mitochondrial physiology between these patients.

Our hypothesis was that a change in walking performance has a relationship to changes in mitochondrial function and negative responders decrease while positive responders increase mitochondrial capacity.

## Methods

### Patient screening

A total of 28 patients limited by intermittent claudication were enrolled into this study. The patients were recruited between February 2015 and January 2016 at the Department of Vascular Surgery, St Olavs University Hospital, Trondheim, Norway. All experimental protocols and procedures were approved by the regional committee of medical and health research ethics, central Norway (nr. 2011/2533) and conformed to the Declaration of Helsinki. Written informed consent was obtained from all participants. The study was registered in ClinicalTrials.gov (ID: NCT 023110256).

The patients were physically examined and interviewed during a baseline visit. They were included by the following criteria: (1) a history of intermittent claudication, (2) walking ability limited by intermittent claudication during a screening treadmill test, (3) an ankle brachial index (ABI) at rest between 0.4 and 0.9. No age restrictions were applied. Patients with vascular interventions more than 3 months ago independent of indication and still having intermittent claudication, were eligible for inclusion.

Patients were excluded when having (1) absence of peripheral arterial disease (PAD), (2) asymptomatic PAD, (3) critical limb ischemia; defined as those with ABI below 0.4 combined with rest pain or ischemic ulcer, (4) exercise tolerance limited by factors other than claudication (e.g., coronary artery disease, dyspnea), (5) vascular interventions in the last 3 months, (6) usage of antiplatelet drugs other than acetylsalicylic acid (e.g., Plavix, Persantine), (7) usage of anticoagulants (e.g., Warfarin), (8) diabetes mellitus, (9) active cancer and (10) renal- or liver disease which needed treatment or follow up.

### Procedures

Patients were evaluated at baseline and after 8 weeks of the exercise intervention. During each visit, patients completed tests in the following order: (1) physical examination including a review of current medication (2) collection of muscle biopsies (3) 6-min walk test (4) treadmill testing (5) peripheral hemodynamics tests.

At the end of the baseline visit participants were randomized between a calf raise exercise group (n = 14) or a walking exercise group (n = 14). An internet-based randomization database using a 1:1 allocation, offered by the Unit for Applied Clinical Research at our Medical Faculty was used. Assessors were blinded to the allocation of each participant. We randomized between two exercise programs in order to assure similar clinical characteristics in the groups.

Our intention was to see whether different types of responders in walking performance and mitochondrial capacity exist, independent of type of exercise. Thereby focusing on the individual response and not the group to which an individual belongs to.

### Exercise interventions

A calf raise exercise group was instructed to perform calf raise exercise three times a day. Calf raise exercise consisted of the subject standing in front of a wall, which was used for support of the balance. The body was lifted using the calf muscles to the maximal height that the subject could achieve. This was repeated until pain was felt in the calf musculature. Following initiation of pain, the subject was instructed to perform five extra calf raises. The five extra calf raises after pain secured ischemia followed by reperfusion of the muscle at rest. Thereby establishing a preconditioning situation.

A walking exercise group was instructed to walk near the pain threshold for 30 min, three times a week. Both training regimens were home-based and without supervision for 8 weeks. No instructions were given on risk factor management or lifestyle modification to any of the groups.

### Measurements

#### Walking performance and oxygen uptake

##### Six-minute walk test

A standardised protocol was followed [13]. The self-paced 6-min walk test assessed the pain free walking distance as the distance at which participants first reported pain and the distance covered during 6 min.

Maximal walking distance was the distance at which participants stopped because of claudication pain.

##### Treadmill testing

A treadmill (Woodway, USA) together with the device METAMAX II and the METASOFT software (Cortex, Germany) was used for cardio pulmonary treadmill testing. A graded Gardner-Skinner protocol [14] was applied; the treadmill was at a constant speed of 3.2 km/ h, starting at 0% inclination. The inclination increased by 2% every second minute until the end of the test. Peak oxygen uptake (VO_2peak_ = ml/kg/min) was determined as being the highest value obtained.

Claudication onset time was the onset of claudication pain and peak walking time was the time at which participants stopped the test, during the tests on the treadmill.

#### Subgroups of negative and positive responders

Peak walking time on the treadmill measures maximal walking performance and was used to differentiate between subgroups of negative and positive responders. Negative responders (n = 8) and positive responders (n = 8) were defined as those that decreased or increased peak walking time the most, independent of exercise regiments.

#### Peripheral hemodynamics

##### Ankle brachial index

Ankle brachial index, an evaluation of blood pressures in the arm and the ankle was performed at rest [15]. The systolic pressure of the posterior tibial artery was first measured and thereafter in the dorsalis pedis artery in all patients. We first measured in the most healthy leg and then in the most sick leg, according to the patients complaints. The highest pressure at the ankle was divided by the highest brachial systolic pressure of two measurements to form the ankle-brachial index (ABI) for each leg.

##### Plethysmography

Plethysmograph assessments were conducted as previously described [16]. The blood flow in the lower extremity was assessed with a strain-gauge plethysmography (Hokanson A16 Inc, Bellevue). A strain gauge was placed around the widest girth of the calf. A thigh cuff was inflated to 220 mmHg for 5 min, to induce ischemia followed by hyperemic reperfusion to the leg. During the last minute of arterial occlusion, an ankle cuff was inflated to 250 mmHg, to exclude the blood circulation to and from the foot. The thigh cuff was deflated but during ten measurements the thigh cuff was again inflated for each measurement at 40 mmHg to block the venous reflux. Thereby the arterial inflow to the calves was measured as a maximal hyperemic response (ml/min/100 ml of calf volume).

##### Muscle biopsy

Biopsies were collected at baseline and after 8 weeks from the lateral part of the gastrocnemius muscle. A micro biopsy technique was conducted to obtain muscle tissue [17]. The sampling site was shaved and the skin was sterilized with chlorhexidine 5% and anesthetized by subcutaneous injection of xylocain with adrenalin (Astra Zeneca, Oslo, Norway). The local anesthetic was strictly injected in the subcutaneous tissue, to avoid influence of the muscle mitochondria. A 14-gauge insertion cannula (BioPince, Medical device technologies Inc., Gainesville, Florida USA) punctured the skin perpendicular until the fascia was pierced. A sterile 16-gauge biopsy needle was introduced through the cannula and muscle biopsy samples were obtained from the gastrocnemius muscle.

##### Permeabilized skeletal muscle fiber preparation

The muscle tissue was immediately transferred into ice-cold biopsy preservation solution (BIOPS) containing 10 mM Ca-EGTA buffer, 0.1 μM free calcium, 20 mM imidazole, 20 mM taurine, 50 mM 2-(N-morpholino) ethane-sulfonic acid hydrate, 0.5 mM dithiothreitol, 6.56 mM MgCl_2_, 5.77 mM ATP, 15 mM phosphocreatine (pH 7.1) [18]. A sample of the muscle tissue was transferred into a small petri dish filled with BIOPS and placed on ice. Muscle samples were gently dissected using forceps and fibers were chemically permeabilized via incubation in two ml of BIOPS containing saponin (50 μg/ml) for 30 min. The objective was to permeabilize the extracellular membranes of the muscle fibers leaving intracellular membranes of the mitochondria intact. The muscle fibers were then washed for 10 min at 4 °C in a mitochondrial respiration medium (MiR05) containing 110 mM sucrose, 60 mM K^+^-lactobionate, 0.5 mM EGTA, 3 mM MgCl_2_, 20 mM taurine, 10 mM KH_2_PO_4_, 20 mM HEPES and 1 g/l bovine serum albumin (pH 7.1). The wet weight of muscle fibers (1–3 mg) was measured on a microbalance (Sartorius ME235P-SD; Sartorius AG, Goettingen, Germany) immediately before assessment of mitochondrial respiration.

##### Mitochondrial respirometry

The muscle fibers were transferred into a 2 ml glass chamber containing MiR05 for high-resolution respirometry measurements (Oxygraph-2 k; Oroboros Instruments, Innsbruck, Austria). The oxygen concentration and oxygen consumption were continuously recorded in the chamber. Oxygen consumption per second, per milligram of wet weight of muscle fibers was addressed as mitochondrial respiration (pmol O_2_/s/mg wet weight of muscle fibers). Measurements were performed at 37 °C. All experiments were carried out in hyper-oxygenated chambers (250–500 μM oxygen) to prevent any potential oxygen limitations. The Substrate, Uncoupler and Inhibitor Titration (SUIT) protocol was used to examine different branches of the electron transfer system as previously described [18–20]. This was achieved by adding inductive or blocking substrates to the chamber. All respirometric analyses were made in duplicates. The LEAK state (presented with subscript _L_) represents the resting mitochondrial respiration of an unaltered and intact electron transport system in the absence of ADP. The LEAK state is measured from the electron flow through complex I (CI) and electron transferring flavoprotein (ETF). ETF linked transfer of electrons was induced with the addition of octanoylcarnitine (0.2 mM) and malate (2 mM). Malate was added because the ETF linked transfer of electrons requires the metabolism of acetyl-CoA to facilitate convergent electron flow into the Q-junction from both complex I (CI) and ETF (ETF + CI)_L_.

OXPHOS state (presented with subscript _P_) represents maximal electron flow through the electron transfer system in the presence of ADP. Electron transferring-flavoprotein capacity (ETF + CI)_P_ was determined following the addition of ADP (5 mM). Mitochondrial respiration specific to complex I (CI + ETF)_P_ was induced by the addition of glutamate (10 mM). Respiration supported by complex I and complex II (CI + CII + ETF)_P_, was then induced with the addition of succinate (10 mM). (CI + CII + ETF)_P_ demonstrates a naturally intact electron transport system’s capacity to catalyze a sequential set of redox reactions that are partially coupled to the production of ATP at complex V [18].

Electron transfer system (presented with subscript _E_) state represents the electron transport through the electron transfer system, when it is uncoupled from ATPase (complex V). Electron transfer system capacity (CI + CII + ETF)_E_ was assessed through titration of the proton ionophore, carbonyl cyanide p-(trifluoromethoxy) phenylhydrazone (FCCP: 0.5 μM stepwise titration to optimum concentrations ranging from 1.5 to 3 μM). Rotenone (0.5 μM) was added to inhibit complex I, thereby electron flow specific to complex II (CII)_E_ can be measured. Finally, malonic acid (5 mM) and antimycin A (2.5 μM) were added, to terminate respiration by inhibiting complex II and complex III respectively.

### Mitochondrial quality and control

The OXPHOS coupling efficiency, the excess electron transfer system-phosphorylation factor capacity and complex II control factor were calculated as measures of mitochondrial quality and control. OXPHOS coupling efficiency, calculated with the formula (1 − (ETF + CI)_L_/(ETF + CI)_P_), reflects the coupling of respiration supported by electron transferring flavoprotein (ETF) with octanoylcarnitin and malate as substrates before (ETF + CI)_L_ and after addition of ADP (ETF + CI)_P_. The OXPHOS coupling efficiency (OCE) can be compared with the respiratory control ratio (RCR). The formula of OCE could be rewritten as; ${\text{OCE }} = \left( {1 - \frac{1}{RCR}} \right)$. The OCE is noted between 0 and 1, while the RCR could be from 0 to infinite. A lower value of OCE means lesser coupling of the oxidation and phosphorylation after addition of ADP. As the OCE decreases it is therefore less coupled.

The excess electron transfer system-phosphorylation factor capacity (1 − (CI + CII + ETF)_P_/(CI + CII + ETF)_E_) is an expression of the relative limitation of OXPHOS capacity (CI + CII + ETF)_P_ by the electron transport system capacity (CI + CII + ETF)_E_ of the phosphorylation system.

The complex II control factor (1 − (CI + ETF)_P_/(CI + CII + ETF_P_)) reflects the fractional change of mitochondrial respiration when succinate (CI + CII + ETF)_P_ is added to respiration supported by complex I (CI + ETF)_P_.

### Mitochondrial content

Citrate synthase activity was used as a biomarker mitochondrial content [21]. Citrate synthase activity (μmol/min/mg of protein) was assayed in homogenates of the permeabilized fibers used in the respiration measurements [19]. The content of the chambers was removed after each respiration experiment and washed with 0.2 ml MiR05 for 10 min at 4°. The fluid was frozen at minus 80°. Citrate synthase activity was measured later with a spectro-photometer at 412 nm and 25 °C (Citrate Synthase Assay Kit, Sigma-Aldrich), according to the manufacturer’s instructions.

### Statistical analyses

Multiple linear regression of the whole group, including both exercise regimens, (n = 28) was used to identify whether changes in mitochondrial capacity were related to changes in walking performance. Baseline ankle brachial index, acetylsalicylic acid usage, smoking, hypertension and previous vascular surgery were used as covariates. Unpaired Students *t* test was used to compare baseline clinical characteristics and mitochondrial capacity between subgroups. Paired-t-test and analysis of covariance (ANCOVA) were used to compare changes in mitochondrial capacity within each group, and in comparison between subgroups. Statistical significance was considered at a value of p < 0.05.

## Results

### Participants

Among the positive responders 3 and in the negative responders 5 out of 8 originated from the walking group. The subgroups differed; negative responders had more often previous vascular surgery and higher blood pressure at baseline. This might indicate a more severe atherosclerotic disease (Table 1).

**Table 1:** Baseline clinical data

	Whole group (n = 28)	Positive responders (n = 8)	Negative responders (n = 8)	Comparison between subgroups
	Mean ± SE	Mean ± SE	Mean ± SE	p value
Sex (female)	13 (44)	4 (50)	4 (50)	1.00
Age	69.2 ± 1.7	67.3 ± 3.45	69.3 ± 3.35	0.95
BMI	26.2 ± 0.7	26.6 ± 1.3	26.7 ± 0.9	0.95
Anamnestic walking distance (m)	348 ± 54	333 ± 120	194 ± 33	0.15
Pain free walking distance (m)	240 ± 33	178 ± 34	310 ± 100	0.12
Six minutes walking distance (m)	460 ± 10	448 ± 24	456 ± 21	0.33
Maximal walking distance (m)	591 ± 42	511 ± 46	561 ± 82	0.39
Claudication onset time (s)	270 ± 28	217 ± 50	294 ± 49	0.15
Peak walking time (s)	648 ± 61	667 ± 161	620 ± 81	0.40
VO_2_ peak (ml/kg/min)	18 ± 0.7	18 ± 2	18 ± 1	0.99
Maximal blood flow (ml/100 ml/min)	10.9 ± 0.9	10 ± 0.5	9.96 ± 2.4	0.96
Ankle brachial index	0.57 ± .0.03	0.54 ± 0.05	0.52 ± 0.04	0.76
Smoking	7 (25)	1 (13)	4 (50)	0.05
Aspirin	20 (71)	6 (75)	4 (50)	0.15
Statin	17 (60)	5 (62)	3 (38)	0.16
Previous vascular surgery	8 (29)	0 (0)	5 (62)	0.03
Systolic pressure	144 ± 4	134 ± 7	156 ± 10	0.04
Diastolic pressure	82 ± 2	77 ± 4	89 ± 3	0.01

p values are calculated by *t* test for continuous variables and Chi square test for categorical variables between sub-group of negative and positive responders. Data are mean ± standard error of the mean (SE) for continuous variables, and numbers (proportions; %) for categorical variables

### Changes in walking performance after exercise

**Fig. 1:**
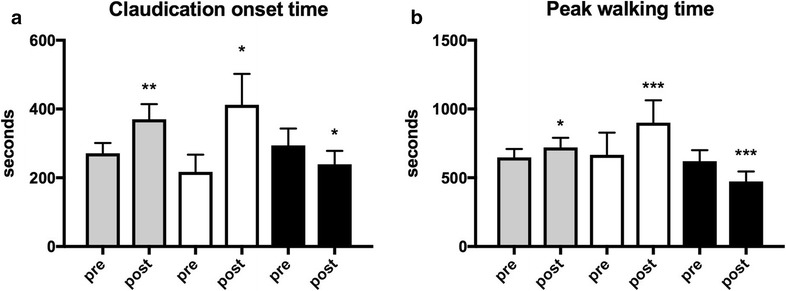
Treadmill walking performance in the whole group (n = 28, *grey*) and subgroups of positive (n = 8, *white*) and negative responders (n = 8, *black*) **a** claudication onset time. **b** peak walking time. Values are mean and standard error of the mean. p values are calculated by paired t test within groups and analysis of covariance (ANCOVA) between subgroups of negative and positive responders. *(p < 0.05), **(p < 0.01), ***(p < 0.001)

Claudication onset time and peak walking time increased in the whole group (p = 0.0058, p = 0.0144), in the positive responders (p = 0.0273, p < 0.00001) and decreased in the negative responders (p = 0.049, p = 0.0005) (Fig. 1).

### Peripheral hemodynamics

Ankle brachial index (ABI) and maximal hyperemic response to ischemia did not change within groups and did not differ between groups.

### Relationship between peak walking time and mitochondrial function

Multiple linear regression of the whole group (n = 28) showed a relationship between changes in peak walking time and changes in mitochondrial respiration supported by electron transferring flavoprotein ((ETF + CI)_P_, p = 0.004), complex I ((CI + ETF)_P_, p = 0.003), complex I and II ((CI + CII + ETF)_P_, p = 0.037) and OXPHOS coupling efficiency (p = 0.046) (Table 2).

**Table 2:** Relationship between peak walking time and mitochondrial respiration, quality, control and content after exercise in the whole group (n = 28)

	β-Coefficient	SE	95% confidence interval	p value	
(ETF + CI)_L_	−10.02	5.08	−20.74	0.70	0.065
(ETF + CI)_P_	10.54	3.14	3.92	17.15	0.004
(CI + ETF)_P_	12.30	3.57	4.77	19.84	0.003
(CI + CII + ETF)_P_	4.77	2.10	0.33	9.20	0.037
(CI + CII + ETF)_E_	2.41	1.45	−0.66	5.47	0.116
(CII)_E_	3.49	2.10	−0.94	7.92	0.115
OXPHOS coupling efficiency	149	60	20	278	0.046
Excess E-P capacity factor	−127	372	−912	657	0.736
Complex II control factor	−290	365	−1061	480	0.438
Citrate synthase activity	−132	428.	−1065	800	0.762

p values are calculated by multiple linear regression using change in peak walking time as dependent variable and changes in mitochondrial values as the independent values. Baseline ankle brachial index, acetylsalicylic acid usage, smoking, hypertension and previous vascular surgery were used as covariates

Mitochondrial respiration (pmol O_2_/s/mg wet weight of muscle fibers) variables: (ETF + CI)_L_ is the LEAK state electron transfer through electron transferring flavoprotein (ETF) and complex I after addition of the substrates octanoylcarnitin (0.2 mM) + malate (2 mM), in the absence of ADP; (ETF + CI)_P_ is fatty acid OXPHOS capacity after addition of ADP (5 mM); (CI + ETF)_P_ is electron transfer through complex I and ETF reaching complex I OXPHOS capacity after addition of glutamate (10 mM); (CI + II + ETF)_P_ is electron transfer through complex I, II and ETF reaching complex I and II OXPHOS capacity after addition of succinate (10 mM); (CI + II + ETF)_E_ is electron transfer through complex I, II and ETF reaching ETS capacity after FCCP titrations (0.5 M max. 3 M) to uncouple oxidation from phosphorylation; (CII)_E_ is ETS capacity supported by complex II after addition of rotenone (0.5 M), which inhibits complex I. The subscripts L, P, E indicate the LEAK state, OXPHOS and ETS capacity

Variables of mitochondria quality, control and content: OXPHOS coupling efficiency (1 − (ETF + CI)_L_/(ETF + CI)_P_). excess electron transfer system-phosphorylation (E-P) capacity factor (1 − (CI + CII + ETF)_P_/(CI + CII + ETF)_E_). Complex II control factor (1 − (CI + ETF)_P_/(CI + CII + ETF)_P_). Citrate synthase activity (umol/min/mg of protein)

### Mitochondrial respiration

Mitochondrial respiration was similar at baseline in the whole group and subgroups.

**Fig. 2:**
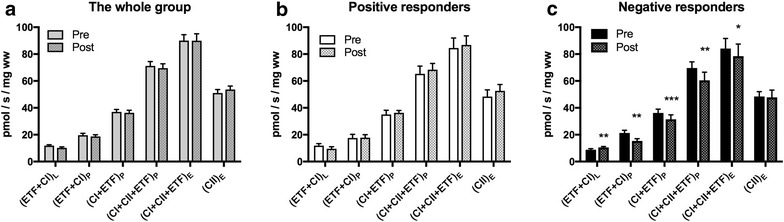
Mitochondrial respiration. For explanations of the variables see legend Table 2. **a** Whole group (n = 28). **b** Positive responders (n = 8). **c** Negative responders (n = 8). Values are mean and standard error of the mean. p values are calculated by paired *t* test within groups and analysis of covariance (ANCOVA) between subgroups of negative and positive responders. *(p < 0.05), **(p < 0.01), ***(p < 0.001)

Mitochondrial respiration did not change in the whole group and the subgroup of positive responders (Fig. 2a, b). Negative responders decreased mitochondrial respiration supported by electron transferring flavoprotein (ETF + CI)_P_ (p = 0.0013), complex I (CI + ETF)_P_ (p = 0.0005), complex I + complex II (CI + CII + ETF)_P_ (p = 0.011) and electron transfer capacity (CI + CII + ETF)_E_ (p = 0.021). Furthermore they increased mitochondrial respiration supported by electron transferring flavoprotein (ETF + CI)_L_ (p = 0.0058) before addition of ADP (Fig. 2c). The latter indicating a pathological dyscoupling in mitochondrial respiration.

### Mitochondrial quality, control and mitochondrial content

**Fig. 3:**
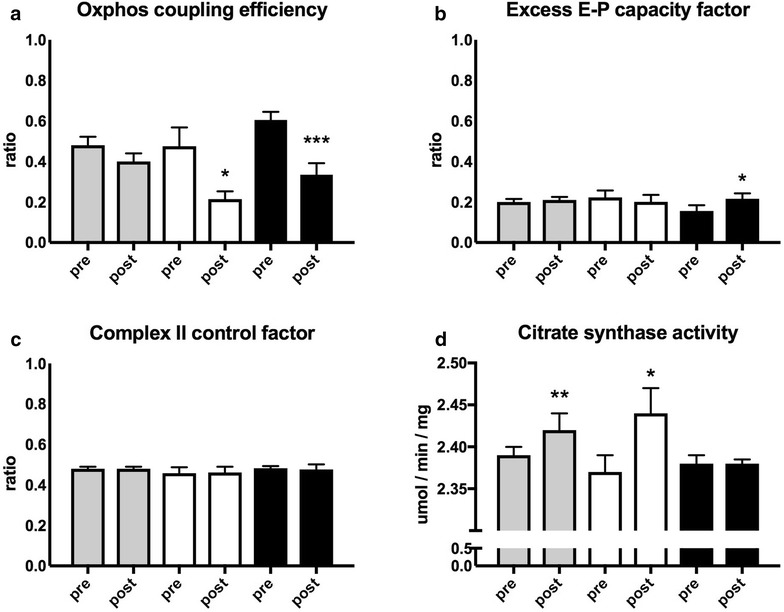
Mitochondrial quality, control and mitochondrial content. Treadmill walking performance in the whole group (n = 28, *grey*) and subgroups of positive (n = 8, *white*) and negative responders (n = 8, *black*). **a** OXPHOS coupling efficiency (1 − (ETF + CI)_L_/(ETF + CI)_P_). **b** Excess electron transfer system-phosphorylation (E-P) capacity factor (1 − (CI + CII + ETF)_P_/(CI + CII + ETF)_E_). **c** Complex II control factor (1 − (CI + ETF)_P_/(CI + CII + ETF)_P_). **d** Citrate synthase activity (umol/min/mg of protein); Values are mean and standard error of the mean. p values are calculated by paired *t* test within groups and analysis of covariance (ANCOVA) between subgroups of negative and positive responders, *(p < 0.05), **(p < 0.01), ***(p < 0.001)

Measures of mitochondrial quality, control and mitochondrial content were similar at baseline in the whole group and subgroups. OXPHOS coupling efficiency decreased in positive (p = 0.045) and negative responders (p = 0.0007) (Fig. 3a). Meaning a decreased coupling of oxidation to phosphorylation, possibly leading to less ATP production, if not compensated for with increased mitochondrial content. Excess electron phosphorylation capacity factor increased in the negative responders (p = 0.03), indicating increased limitation of mitochondrial capacity (CI + CII + ETF)_P_ by the electron transport capacity (CI + CII + ETF)_L_ (Fig. 3b). Citrate synthase activity, a biomarker of mitochondrial content, increased in the whole group (p = 0.008) and the positive responders (p = 0.010), while it did not change in the negative responders (p = 0.90) (Fig. 3d).

### Mitochondrial respiration corrected for mitochondrial content

Mitochondrial respiration was corrected for mitochondrial content by dividing mitochondrial respiration values with citrate synthase activity.

**Fig. 4:**
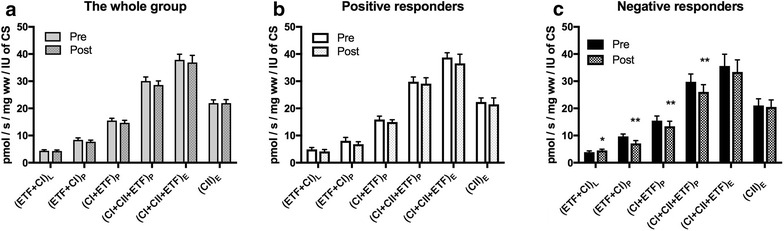
Mitochondrial respiration corrected for mitochondrial content. **a** Whole group (n = 28). **b** Positive responders (n = 8). **c** Negative responders (n = 8). For explanations of the variables see legend Table 2. Values are mean and standard error of the mean. p values are calculated by paired t-test within groups and analysis of covariance (ANCOVA) between subgroups of negative and positive responders. *(p < 0.05), **(p < 0.01)

After this calculation, corrected mitochondrial respiration did not change in the whole group and the subgroup of positive responders (Fig. 4a, b). While the negative responders decreased corrected mitochondrial respiration supported by electron transferring flavoprotein (ETF + CI)_P_ (p = 0.0017), complex I (CI + ETF)_P_ (p = 0.0044), complex I + complex II (CI + CII + ETF)_P_ (p = 0.0046) and electron transfer capacity (CI + CII + ETF)_E_ (p = 0.0005) (Fig. 4c).

## Discussion

This study shows that changes in walking performance after exercise relate to changes in mitochondrial capacity. We also demonstrate that exercise induces mitochondrial changes that seem to be different in subgroups of negative and positive responders. Negative responders decreased mitochondrial respiration after 8 weeks of exercise, while positive responders increased citrate synthase activity. Surprisingly no increase in mitochondrial respiration was found in the positive responders.

The variable peak walking time was used to differentiate between negative and positive responders. Peak walking time is achieved at maximal effort of patients with IC during a standardized treadmill test. Extensive experience in multicentre studies with treadmill testing has been acquired over several decades and is therefore preferred over other tests of walking performance [22].

Previous research supports that exercise, independent of type and intensity, is beneficial for walking performance in patients with IC [23]. In our study exercise induced improvements in walking performance in certain patients and reduced or had no effect in others. Even if previous group analyses demonstrate the benefit of exercise, this might not be the case for each individual. The present paper is an example of an approach, which tries to establish personalised medicine. We try to define what is good for a single patient and not based on what is good for the patient according to the group he or she belongs to.

Several exercise studies on patients with IC have shown that mitochondrial enzyme activities increase after exercise. Relationship between increases in mitochondrial enzymes cytochrome C oxidase (complex IV) [4] and succinic oxidase (complex II) [24] and improvements in walking performance seem to exist. Lundgren et al. observed that an increase in cytochrome C oxidase was related to increased walking performance after exercise [4]. We used mitochondrial respirometry, which is an assay of complexes collectively functioning during oxidative phosphorylation in actively respiring muscle mitochondria. The mitochondrial enzyme activities, as used by Lundgren et al., are assays of one complex isolated from the other complexes, which does not reflect the whole mitochondrial physiology. Based on our data we also show that a relationship between changes in walking performance and changes in mitochondrial respiration supported by electron transferring flavoprotein and complex I seem to exist.

The negative responders seemed to have a more advanced degree of peripheral arterial disease since they had a higher blood pressure and more often had been subjected to vascular surgery. This is in line with Dörenkamp et al. who show that changes in pain free walking time after exercise were related to cardiac morbidity [25]. Both the data of Dörenkamp et al. and ours indicate that the response to exercise is dependent on degree of arteriosclerosis. Dörenkamp et al. also showed that age, gender, BMI and current smoking influenced walking performance after exercise. These variables were not different in our subgroups. This could be a result of the small number in our study. Some clinical variables connected to atherosclerosis might therefore predict the outcome following exercise but according to our data not the state of the mitochondria at baseline.

Similar levels of mitochondrial respiration and citrate synthase activity were found in the subgroups at baseline. The response to exercise could thereby be compared based on the change within and between each subgroup. After 8 weeks, the negative responders decreased mitochondrial respiration mainly supported by electron transferring protein (ETF) and complex I. Furthermore, a decreased OXPHOS coupling efficiency (OCE) was found in negative responders suggesting a less coupled electron transfer system. The formula for calculating OCE is (1 − (ETF + CI)_L_/(ETF + CI)_P_) which represents the ratio of oxygen consumption (supported by ETF and CI) from ADP to ATP. Therefore, the formula could be rewritten as OCE = (1 − before ADP/after ADP). OCE is an indirect measurement of the ADP/ATP ratio. The direct measurement of the ADP/ATP ratio is generally used as a marker of the energy state of the cell and apoptosis. Low ADP/ATP ratios indicate a low energy state of the cell and is expected in an electron transfer system when OCE is low. A poor efficiency of the electron transfer system to produce ATP from ADP might cause a low ADP/ATP ratio.

The excess electron transfer system-phosphorylation factor capacity was increased and thereby the mitochondrial capacity is limited by the electron transfer system capacity. Altogether we demonstrate reduced mitochondrial capacity and increased mitochondrial dysfunction in negative responders after exercise.

Increased mitochondrial DNA injury has been shown in peripheral arterial disease [26–28]. Respiration supported by ETF and complex I decreased in the negative responders, while complex II did not change. ETF and complex I are encoded by both nuclear and mitochondrial DNA, while complex II is encoded only by nuclear DNA. This supports the notion that damage to mitochondrial DNA might partly be the cause of decreased mitochondrial respiration in the negative responders.

Interestingly mitochondrial respiration was similar in positive responders at baseline and after the exercise intervention indicating that mitochondrial DNA might be preserved. Positive responders only increased citrate synthase activity. Positive responders seem to improve mitochondrial capacity through increased quantity possibly compensating for the reduced OXPHOS coupling efficiency. Thereby being able to utilize more ATP for a given amount of oxygen, which results in a longer peak walking time.

Mitochondrial respiration has been shown to be preserved after ischemic preconditioning [9, 29]. While reduced mitochondrial respiration has been shown with increased degree of peripheral arterial disease [1]. Probably both nuclear and mitochondrial DNA play a central role in the response to ischemia and reperfusion. ROS and mapping of nuclear and mitochondrial genome of patients with IC might be the next approach to personalized medicine for these patients. The goal being to predict whether improvements after exercise are likely to occur in each patient individually. Future research in our group will elucidate whether exercise of patients with intermittent claudication increase mitochondrial ROS production and whether this results in damage to mitochondrial DNA, dependent on the level of disease.

## Conclusions

Negative responders to exercise, measured as decreased peak walking time, have a more advanced degree of peripheral arterial disease compared to those that respond positively. The negative responders reduce their mitochondrial respiration capacity, whereas positive responders improve mitochondrial capacity after exercise, through increased mitochondrial content. Furthermore, relationships between changes in peak walking time and mitochondrial respiration seem to exist.

## Limitation

The training regimens were home-based and without supervision, which could be a leading factor to variability. It is normal practice at the vascular clinic that instruction in exercise is only provided once to patients, and it was the intent of the current study to reproduce this practice. We were mainly interest in the effects after one single instruction of exercise and not the effect of different types of exercise. The latter would have needed supervision to guarantee adherence.

## Abbreviations

ABI: ankle brachial index
ETF: electron transferring flavoprotein
ETS: electron transfer system
IC: intermittent claudication
OXPHOS: oxidative phosphorylation
PAD: peripheral arterial disease
ROS: radical oxygen species
SE: standard error of the mean

## References

[1] Pipinos II, Sharov VG, Shepard AD, Anagnostopoulos PV, Katsamouris A, Todor A (2003). Abnormal mitochondrial respiration in skeletal muscle in patients with peripheral arterial disease. J Vasc Surg.

[2] Brass EP, Hiatt WR (2000). Acquired skeletal muscle metabolic myopathy in atherosclerotic peripheral arterial disease. Vasc Med.

[3] Parmenter BJ, Raymond J, Dinnen P, Singh MAF (2011). A systematic review of randomized controlled trials: walking versus alternative exercise prescription as treatment for intermittent claudication. Atherosclerosis.

[4] Lundgren F, Dahllöf AG, Scherstén T, Bylund-Fellenius AC (1989). Muscle enzyme adaptation in patients with peripheral arterial insufficiency: spontaneous adaptation, effect of different treatments and consequences on walking performance. Clin Sci.

[5] Gardner AW, Poehlman ET (1995). Exercise rehabilitation programs for the treatment of claudication pain. A meta-analysis. JAMA.

[6] Norgren L, Hiatt WR, Dormandy JA, Nehler MR, Harris KA, Fowkes FGR (2007). Inter-society consensus for the management of peripheral arterial disease (TASC II). Eur J Vasc Endovasc Surg.

[7] Kooijman HM, Hopman MT, Colier WN, van der Vliet JA, Oeseburg B (1997). Near infrared spectroscopy for noninvasive assessment of claudication. J Surg Res.

[8] Slagsvold KH, Johnsen AB, Rognmo O, Høydal MA, Wisløff U, Wahba A (2014). Mitochondrial respiration and microRNA expression in right and left atrium of patients with atrial fibrillation. Physiol Genom.

[9] Thaveau F, Zoll J, Rouyer O, Chafke N, Kretz JG, Piquard F (2007). Ischemic preconditioning specifically restores complexes I and II activities of the mitochondrial respiratory chain in ischemic skeletal muscle. J Vasc Surg.

[10] Tisi PV, Hulse M, Chulakadabba A, Gosling P, Shearman CP (1997). Exercise training for intermittent claudication: does it adversely affect biochemical markers of the exercise-induced inflammatory response?. Eur J Vasc Endovasc Surg.

[11] Tisi PV, Shearman CP (1998). The evidence for exercise-induced inflammation in intermittent claudication: should we encourage patients to stop walking?. Eur J Vasc Endovasc Surg.

[12] Larsen OA, Lassen NA (1966). Effect of daily muscular exercise in patients with intermittent claudication. Lancet.

[13] ATS Committee on Proficiency Standards for Clinical Pulmonary Function Laboratories (2002). ATS statement: guidelines for the 6-min walk test. Am J Respir Crit Care Med.

[14] Hiatt WR, Regensteiner JG, Hargarten ME, Wolfel EE, Brass EP (1990). Benefit of exercise conditioning for patients with peripheral arterial disease. Circulation.

[15] McDermott MM, Liu K, Guralnik JM, Mehta S, Criqui MH, Martin GJ (1998). The ankle brachial index independently predicts walking velocity and walking endurance in peripheral arterial disease. J Am Geriatr Soc.

[16] Alomari MA, Solomito A, Reyes R, Khalil SM, Wood RH, Welsch MA (2004). Measurements of vascular function using strain-gauge plethysmography: technical considerations, standardization, and physiological findings. Am J Physiol Heart Circ Physiol.

[17] Hayot M, Michaud A, Koechlin C, Caron M-A, LeBlanc P, Prefaut C (2005). Skeletal muscle microbiopsy: a validation study of a minimally invasive technique. Eur Respir J.

[18] Pesta D, Gnaiger E. High-resolution respirometry: OXPHOS protocols for human cells and permeabilized fibers from small biopsies of human muscle. In: Moreno AJ, Palmeira CM, editors. Totowa: Humana Press; 2012. p. 25–58. 10.1007/978-1-61779-382-0_3 22057559

[19] Jacobs RA, Lundby C (2013). Mitochondria express enhanced quality as well as quantity in association with aerobic fitness across recreationally active individuals up to elite athletes. J Appl Physiol.

[20] van Schaardenburgh M, Wohlwend M, Rognmo O, Mattsson EJR (2016). Mitochondrial respiration after one session of calf raise exercise in patients with peripheral vascular disease and healthy older adults. PLoS ONE.

[21] Larsen S, Nielsen J, Hansen CN, Nielsen LB, Wibrand F, Stride N (2012). Biomarkers of mitochondrial content in skeletal muscle of healthy young human subjects. J Physiol.

[22] Hiatt WR, Rogers RK, Brass EP (2014). The treadmill is a better functional test than the 6-minute walk test in therapeutic trials of patients with peripheral artery disease. Circulation.

[23] Lane R, Ellis B, Watson L, Leng GC (2014). Exercise for intermittent claudication. Cochrane Database Syst Rev.

[24] Holm J, Dahllöf AG, Björntorp P, Scherstén T (1973). Enzyme studies in muscles of patients with intermittent claudication. Effect of training. Scand J Clin Lab Invest.

[25] Dörenkamp S, Mesters I, de Bie R, Teijink J, van Breukelen G (2016). Patient characteristics and comorbidities influence walking distances in symptomatic peripheral arterial disease: a large 1-year physiotherapy cohort study. PLoS ONE.

[26] Wang H, Hiatt WR, Barstow TJ, Brass EP (1999). Relationships between muscle mitochondrial DNA content, mitochondrial enzyme activity and oxidative capacity in man: alterations with disease. Eur J Appl Physiol Occup Physiol.

[27] Brass EP, Wang H, Hiatt WR (2000). Multiple skeletal muscle mitochondrial DNA deletions in patients with unilateral peripheral arterial disease. Vasc Med.

[28] Bhat HK, Hiatt WR, Hoppel CL, Brass EP (1999). Skeletal muscle mitochondrial DNA injury in patients with unilateral peripheral arterial disease. Circulation.

[29] Slagsvold KH, Rognmo O, Høydal M, Wisløff U, Wahba A (2014). Remote ischemic preconditioning preserves mitochondrial function and influences myocardial microRNA expression in atrial myocardium during coronary bypass surgery. Circ Res.

